# An Efficient and Easy- to- Use Method for Extraction of *H. pylori* DNA from Archival Formalin-Fixed and Paraffin-Embedded Gastric Tissues

**DOI:** 10.30699/IJP.2023.562113.2974

**Published:** 2023-10-15

**Authors:** Parastoo Saniee, Paria Ghadersoltani, Masoumeh Noroozpour, Alireza Sadjadi

**Affiliations:** 1 *Department of Microbiology and Microbial Biotechnology, Faculty of Life Sciences and Biotechnology, Shahid Beheshti University G.C, Tehran, Iran*; 2 *Digestive Oncology Research Center, Digestive Diseases Research Institute, Shariati Hospital, Tehran University of Medical Sciences, Tehran, Iran*

**Keywords:** DNA extraction, H. pylori, Paraffin-embedded tissue

## Abstract

**Background & Objective:**

Formalin-fixed paraffin-embedded archived tissues are useful for the genetic analyses and assessment of some patients’ disease history, including infectious diseases. However, there is no established protocol for extracting bacterial DNA from the archived specimens. In this study DNA was extracted from the archived *H. pylori*-positive gastric biopsies by some modifications applied to the previously published protocols. The quality of the extracted DNA was assessed by amplifying *H. pylori*-specific* 16S rRNA* gene.

**Methods:**

Fifty *H. pylori*-positive gastric biopsies obtained, fixed, and embedded in paraffin blocks during 2002-2008 were recruited. After paraffin removal, simultaneous proteinase K treatment and mechanical disruption using glass beads were used for the digestion of gastric tissues. DNA extraction was performed by adding one step of phenol treatment and two steps of incubation to the conventional phenol-chloroform method. The quantity and quality of the extracted DNA samples were assessed. Also, PCR was performed using primers specific for the *H. pylori*-specific *16S rRNA*.

**Results:**

The electrophoresis showed that intact DNAs were recovered from all biopsy samples. Amplification of the PCR products with the size of 519bp confirmed the presence of *H. pylori-*specific* 16S rRNA* gene in all the biopsies.

**Conclusion:**

A 100% success rate for the amplification of *H. pylori*-specific *16S rRNA* gene was achieved from all the samples. In this regard, the designed modified method resulted in the effective removal of interfering contaminations and enhanced the quality of the extracted bacterial DNA from the archived tissues. These modifications may contribute to better extraction of the intact DNA from different bacteria present in human tissues.

## Introduction

Formalin-fixed paraffin-embedded (FFPE) tissues obtained as biopsy or excised by surgery, represent valuable sources of the genetic information (1, 2). The main advantage of using such samples would be the possibility of correlating the results of genetic analyses of the patients with their clinical disorders (3, 4). Furthermore, comparing the results of molecular studies on the samples taken from one patient in the past and the present time may provide important data to show the trend of development or severity of the disease (5). Nevertheless, because follow-up clinical information is often collected years after the initial sampling, details of the procedures employed for the preparation and storage of the archived specimens are not easily reachable or may even be unknown (6, 7). Accordingly, the processing of the archived specimens such as extraction of DNA for the molecular studies is not always successful and many valuable samples that were collected many years ago are inevitably excluded (8, 9).

It has been demonstrated that nucleic acid degradation or modifications caused by the protein-nucleic acid interaction during routine histological procedures could limit its application in the subsequent analyses, including polymerase chain reaction (PCR) (10, 11). Regarding FFPE samples, the quality and quantity of the extracted nucleic acids rely on several parameters before, during, and after the extraction. The tissue-cutting conditions and fragment size, block storage time and temperature, fixation, and extraction steps, all are considered as critical issues that may affect the quality of the obtained DNA (12, 13). Several reports demonstrated that even after successful DNA recovery from the FFPE tissues, the amplified products with good quality may not be obtained due to the occurrence of inhibitory residues (14). In this regard, the amplification success rate from the extracted DNA with different methods was estimated as 60–80% (15, 16). Furthermore, it has been reported that only PCR products of up to 100-300 bp were amplified from the FFPE tissues (17, 18).

While most extraction methods have been set up to optimize the recovery of the eukaryotic DNA from the FFPE tissues, there is no established consensus in the literature about the best protocol for extracting bacterial DNA, especially from the archived specimens (19). The long interval between sampling and molecular analyses along with the small size of the target bacterial DNA, compared to the eukaryotic host DNA in tissue samples, could impair the recovery of the intact bacterial DNA and the following amplification steps (20). 

The aim of this study was to detect the *H. pylori* DNA in the paraffin-embedded *H. pylori*-positive gastric biopsies that were archived 14-20 years ago. For DNA extraction from the paraffin blocks, three routine consecutive steps including deparaffinization, tissue digestion, and nucleic acid purification were performed according to the limited published methods (5, 21, 22). Since no *H. pylori-*specific* 16S rRNA* gene was amplified with the used protocols, by some modifications in tissue digestion and DNA purification steps, we could develop an efficient and easy-to-use method for the extraction of bacterial DNA. The quality of extracted DNA was assessed by amplifying *H. pylori*-specific* 16S rRNA* gene.

## Material and Methods

**Formalin-Fixed Paraffin-Embedded (FFPE) Gastric Biopsy Samples**

Fifty gastric biopsies used in this study were obtained from the *H. pylori*-infected patients with positive rapid urease test, during 2002-2008. The biopsies were fixed with formalin and embedded in paraffin blocks according to the standard methods (23, 24) and stored in the Pathology Department, Digestive Disease Research Institute, Tehran, Iran.

**Deparaffinization of Formalin-Fixed Paraffin-Embedded (FFPE) Gastric Biopsy Samples**

Paraffin removal from the FFPE samples was performed according to the method described by Pikor* et al.* (5). The gastric tissue from each paraffin block was separated by a sterile needle and transferred to a 2-ml sterile capped tube. The residual paraffin was dissolved by adding 800 μL of xylene to each tube, followed by the gentle shaking for 10 min. The samples were centrifuged at 13000 rpm for 3 min and the supernatants were discarded. Xylene wash steps were repeated until the paraffin was completely dissolved. The samples were washed with serial dilutions of the absolute ethanol (molecular biology grade, Merck, Germany). Briefly, 800 μL of ethanol was added to each tube, vortexed for 10 sec, and centrifuged at 13000 rpm for 3 min. The supernatants were discarded from tubes and pellets were similarly washed with 70% and then 50% ethanol. After ethanol removal in the last step, the pellets were air-dried for 5 min.

**Digestion of Deparaffinized Gastric Biopsies**

Deparaffinized gastric tissues with an average weight of 15 mg were digested by the protocols described by Mirmomeni *et al.* (21) and Isola *et al.* (22) with some modifications. In our modified method, simultaneous mechanical and enzymatic steps were used for the digestion of gastric tissues. briefly, 50 µL of STES buffer (Tris base 0.2M, NaCl 0.5M, EDTA 0.01M, and SDS 0.1 % w/v), 20 µL of TE buffer (Tris base 10 mM and EDTA 1 mM, pH 7.6) and 20 µL of proteinase K (20 mg /mL) were added to each tube. Then, acid-washed glass beads (10-mg weight) (Sigma, USA) were added to each tube, vortexed vigorously for 1 min, and incubated at 55°C water bath for 24 hr.

**Extraction of DNA from the Gastric Biopsies**

DNA was extracted from the digested tissues using phenol-chloroform method by modifications (25). Equal 60-µL volumes of phenol: chloroform was added to each tube, incubated at 55°C water bath for 1 h, and centrifuged for 5 min at 13000 rpm. The supernatant in each tube was transferred to a new tube. Washing steps were repeated by adding 30 µL of phenol to each tube followed by 10 sec high-speed vortexing, incubation at 55°C water bath for 1 h, and centrifuging for 5 min at 13000 rpm. 

After transferring the supernatant phase to a new tube, 10 µL of sodium acetate (3 M, pH 5.2) and 250 µL of ethanol were added to each tube. Following gentle shaking for 30 sec, tubes were incubated at -20°C for 15 min. The samples were centrifuged for 5 min at 13000 rpm and the supernatants were removed carefully. To precipitate the extracted DNA, 50 µL of 70% ethanol was added to each tube. After centrifuging for 5 min at 13000 rpm, ethanol was removed completely and the samples were air-dried for 20 min. TE buffer in 50 µL (pH 8.3) was added to each tube and the extracted DNAs were used as templates for the PCR reactions. 

**Evaluation of the Extracted DNA**

In order to examine the integrity of the extracted DNAs, all samples were subjected to 1% agarose gel electrophoresis. The quantity and quality of the extracted DNAs were measured in a NanoDrop ND-1000 spectrophotometer (NanoDrop Technologies). The samples with absorbance ratios (A280/A260 nm) between 1.8 and 2 were considered acceptable (12).

**Amplification of** ***H. pylori*** **-Specific** ***16S rRNA*** **Gene from Biopsy Samples**

In order to assess the presence of amplifiable *H. pylori* DNA in total extracted DNAs from gastric biopsy samples, PCR was performed using primers for *H. pylori*-specific *16S rRNA *gene (HP1: GCAATCAGCGTCAGTAATGTTC and HP2: CAAGAGATCAGCCTATGTCC) (26). PCR was performed in a total volume of 25 µL, containing 12.5 µL of PCR master mix (Biofact, Korea), 2 µL DNA (100 ng/µL), and 1 µL of each primer. The amplification steps included: an initial denaturation at 94°C for 3 min, followed by 35 cycles of 94°C for 1 min, annealing at 56°C for 1 min, 72°C for 1 min, and the final extension at 72°C for 5 min. The purified DNA from previously identified *H. pylori* and sterile distilled water were used as positive and negative controls, respectively. The PCR products were visualized using 1% agarose gel and one amplified product was selected to be sequenced with both forward and reverse primers. The sequence homology was determined by the BLAST program.

## Results

**DNA Extraction Results**

**Fig. 1:**
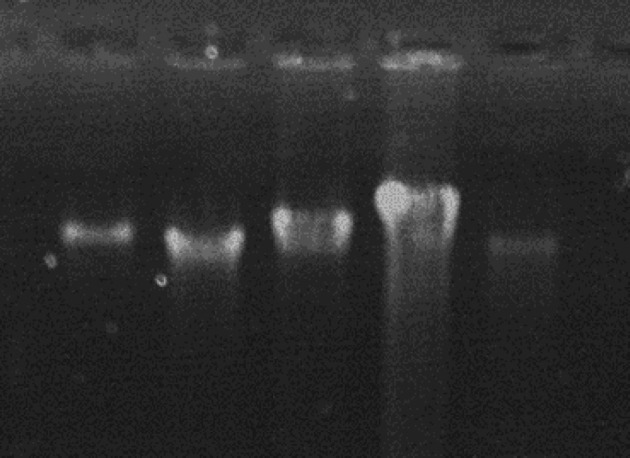
Gel electrophoresis of the extracted DNA from five formalin-fixed and paraffin-embedded gastric tissues on 1% agarose

Electrophoresis of the extracted DNAs showed distinct high molecular weight bands of pure and intact DNAs recovered from the FFPE samples (Figure 1).

Evaluation of the extracted DNAs showed that all 50 samples presented A280/A260 nm values between 1.8 and 2, which were acceptable according to the NanoDrop standard. To assess whether the amplifiable bacterial DNAs were extracted or not, all DNAs were used as PCR templates for the detection of bacterial genes.

**Fig. 2:**
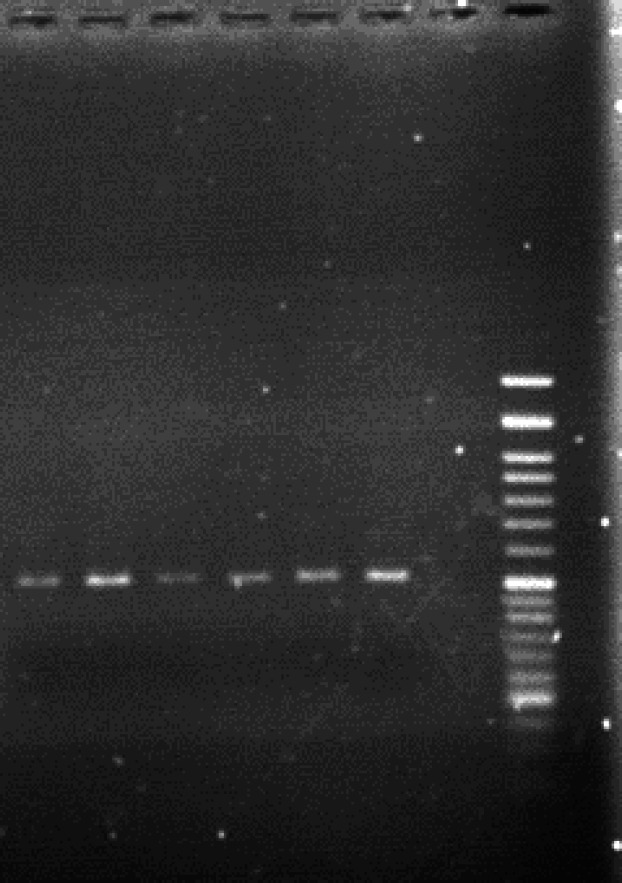
Amplification of the 519 bp fragment of *H. pylori* *16S rRNA* gene from five gastric biopsy samples. Left to right: lane 1-5: PCR products from the gastric biopsies, lane 6: positive control, lane 7: no template, lane 8: molecular ladder

***H. pylori*** **-Specific** ***16S rRNA*** **Gene Amplification from Biopsy Samples**

Amplification of the PCR products with the size of 519bp showed the presence of *H. pylori-*specific* 16S rRNA* gene in all the biopsies (Figure 2). No non-specific bands were visualized. Even in six samples with the ratio of A280/A260 nm below 1.8, the amplified products with high quality were obtained in PCR reactions, indicating that bacterial DNA remained intact in all the samples during the extraction steps.

## Discussion

**Currently, it is evident that bacterial colonization in the human body might be involved in the development of different diseases (** 27 **,** 28 **). The FFPE tissues are an extremely valuable and, in some cases, the only source of the bacterial DNA available for the genetic analysis. While most available protocols focus on extracting the amplifiable eukaryotic DNA from the PFFE tissue, there is no approved protocol for the extraction of the bacterial DNA from this type of specimen** **(** 20 **).**

**In this study, by performing some modifications to the available protocols for DNA extraction, the** ***H. pylori*** **DNA with satisfactory quantity and quality was obtained from all the 50 gastric biopsies that were archived for 14-20 years. DNA extraction from the FFPE tissues was performed according to the previously developed methods that included deparaffinization by xylene, washing in serial dilutions of ethanol, digestion, and finally purification of DNA. Although some authors proposed that deparaffinization is not a required step for the molecular analyses (** 14 **), our results showed no amplification products of** ***H. pylori-16S rRNA*** **gene from DNA that was extracted from paraffinized samples. Accordingly, deparaffinization seems necessary before the process for the effective extraction of the bacterial DNA from the archived samples. While the enzymatic digestion with proteinase K at different concentrations and incubation times has been successful in most of the protocols for digestion of the fresh samples (** 29 **,** 30 **), our results demonstrated that proteinase K alone did not digest the archived tissues completely. A combination of enzymatic treatment and mechanical disruption by glass beads improved the quality of the digested tissues for amplification steps. It appears that mechanical disruption of the cell membrane using glass beads plays a critical role in better extraction of DNA from the old tissues. Furthermore,** **adding one step of phenol treatment and two steps of incubation at 55°C for1 hr in the DNA purification step, compared to the conventional phenol-chloroform method** **, led to an effective removal of any interfering contamination, and enhanced the quality of the extracted bacterial DNA from the FFPE tissues. It has been reported that DNA extracted from the FFPE tissues are suitable for the amplification of the DNA fragments up to only 100-300 bp and usually the PCR reactions fail when longer stretches of DNA templates are targeted (8, 17). Nevertheless, in our study, we could achieve a 100% success rate for the amplification of 519bp fragment of** ***H. pylori*** **-specific** ***16S rRNA*** **gene from all the samples.**

## Conclusion

Extraction of DNA from the old FFPE tissue is a robust procedure and not always successful (21). Furthermore, compared to the human DNA, obtaining bacterial DNA may be more challenging due to the inherently low concentration of bacteria in the biopsy tissues (20). Here, we present a modified method for extraction and purification of bacterial DNA from the archived specimens, which is efficient and easy to handle. The main difference presented in our modified method was for the digestion and extraction steps, which may contribute to better extraction of the intact DNA from different bacteria present in the human tissues.

## Funding

 None.

## Conflict of Interest

The authors declared no conflict of interest.
